# Carbon Nanotube-Doped 3D-Printed Silicone Electrode for Manufacturing Multilayer Porous Plasticized Polyvinyl Chloride Gel Artificial Muscles

**DOI:** 10.3390/gels10070416

**Published:** 2024-06-24

**Authors:** Bin Luo, Hanjing Lu, Yiding Zhong, Kejun Zhu, Yanjie Wang

**Affiliations:** 1 School of Mechanics and Materials, Hohai University, Nanjing 211100, China; yj.wang1985@gmail.com; 2 School of Mechanical and Energy Engineering, Shaoyang University, Shaoyang 422000, China; lhj943164009@163.com (H.L.); zhukejun1098@126.com (K.Z.); 3 State Key Laboratory for Strength and Vibration of Mechanical Structures, Xi’an Jiaotong University, Xi’an 710049, China; ydzhong@zju.edu.cn

**Keywords:** artificial muscle, plasticized PVC gel, direct writing

## Abstract

Plasticized polyvinyl chloride (PVC) gel has large deformation under an applied external electrical field and high driving stability in air and is a candidate artificial muscle material for manufacturing a flexible actuator. A porous PVC gel actuator consists of a mesh positive pole, a planar negative pole, and a PVC gel core layer. The current casting method is only suitable for manufacturing simple 2D structures, and it is difficult to produce multilayer porous structures. This study investigated the feasibility of a 3D-printed carbon nanotube-doped silicone electrode for manufacturing multilayer porous PVC gel artificial muscle. Carbon nanotube-doped silicone (CNT-PDMS) composite inks were developed for printing electrode layers of PVC gel artificial muscles. The parameters for the printing plane and mesh electrodes were explored theoretically and experimentally. We produced a CNT-PDMS electrode and PVC gel via integrated printing to manufacture multilayer porous PVC artificial muscle and verified its good performance.

## 1. Introduction

Artificial muscles exhibit various forms of deformation and output strain and stress under external stimuli such as electricity, magnetic fields, heat, fluid pressure, light, and chemical stimuli. Artificial muscles can function similarly to biological muscle in artificial systems, and they have extensive potential applications in robotics [1,2,3,4,5,6,7,8], flexible electronics [9,10], smart wearables [11], and medical equipment [12,13]. Electroactive polymers (EAPs) are the most practical way to prepare electrically actuated artificial muscles because EAPs have the advantage of being easy to control and have a broad application range [14,15,16,17]. According to working principles, EAP can be classified into two categories: ionic and non-ionic. A typical ionic EAP is an ionic polymer–metal composite (IPMC). Although it has the advantage of a low working voltage of less than 5 V, the electrolyte in it is susceptible to solvent evaporation and is affected by electrolysis [14]. Typical non-ionic EAPs are dielectric elastomers (DEs), which have the advantages of high response frequencies and the ability to work in air, but their working voltages of up to several thousand volts pose the risk of electrical breakdown [15]. Electroactive polyvinyl chloride (PVC) gel, a recent development, has the advantages of large output strain, high output stress, excellent thermal stability, fast response, moderate electric field intensity, low power consumption, and long cycle life [16]. It combines the advantages of the above two representative EAP materials, and thus has become a promising material for electrically actuated artificial muscles.

**Figure 1:**
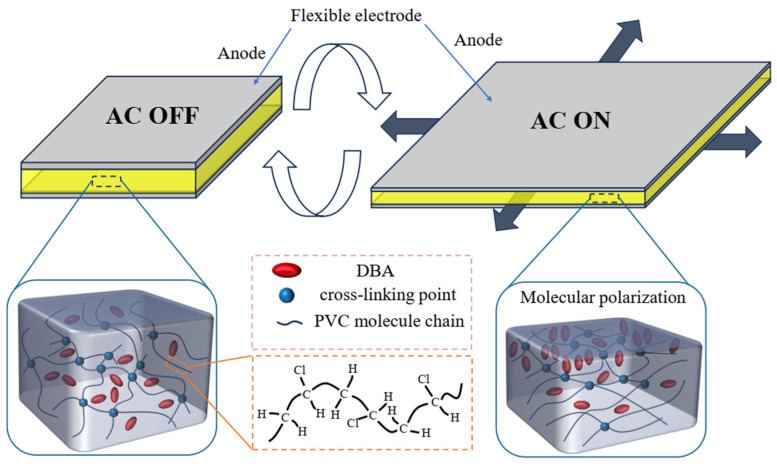
Deformation principle of a PVC gel actuator.

Figure 1 shows the deformation mechanism of a PVC gel structure. Under an electric field, the molecular dipole of the PVC segment rotates and the molecular chain configuration changes. Simultaneously, polarized dibutyl adipate (DBA) molecules migrate to the positive pole, leading to macroscopic creep and deformation of the PVC gel near the positive pole. The artificial muscle returns to its original shape owing to the elasticity of the PVC gel core layer after the electric field is removed [13].

**Figure 2:**
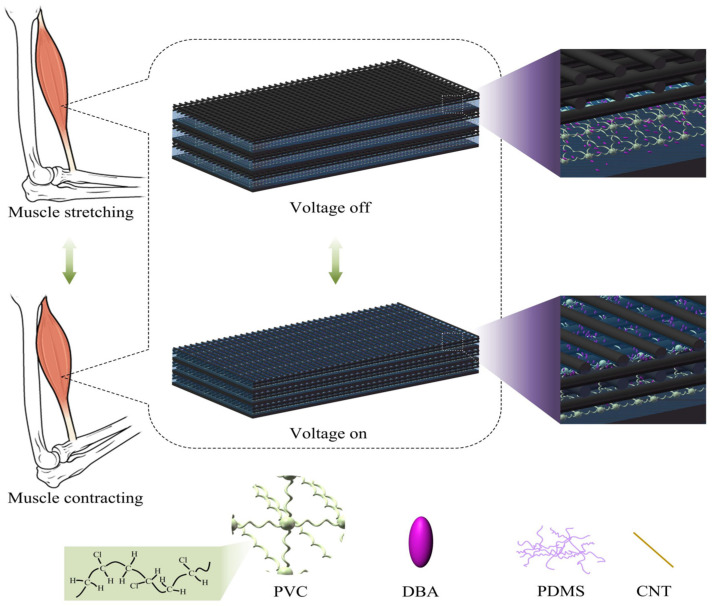
Deformation mechanism and composition of a porous PVC gel actuator.

A porous PVC gel actuator comprises a mesh positive pole, a planar negative pole, and a PVC gel core layer [12]. The pore structures in porous actuators, as depicted in Figure 2, allow PVC gel materials to migrate into the mesh positive pole under an applied electric field, enabling substantial deformation.

The current method for manufacturing porous PVC gel artificial muscles involves pouring a precursor solution, obtained by mixing PVC, DBA, and tetrahydrofuran (THF), into a mold to cast the PVC gel films [11]. After cutting, the PVC gel films and the metal electrodes are manually stacked and packaged to fabricate a PVC gel actuator. However, casting is only suitable for producing simple 2-dimensional structures, and would encounter challenges in manufacturing multilayer porous integrated structures. This can be overcome through additive manufacturing. Helps studied a filament additive manufacturing method that can create complex PVC gel structures. In this method, plastisol was formed by mixing PVC particles with DIDA plasticizer and was processed into PVC gels suitable for additive manufacturing [18]. Nonetheless, actuators consisting of PVC gels require thermoplastic electrodes, while most electrode materials do not meet this requirement. Wang has explored the printing of a PVC core via direct writing, but integrated printing of an electrode and core has not been investigated [19,20]. The research team proposed for the first time the integrated printing process of a shape memory polymer doped with CNT electrodes into the PVC gel core layer. However, due to the lack of thixotropic properties in the memory polymer-doped carbon nanotube electrode ink, it is difficult to achieve the printing of mesh electrodes, making it unsuitable for the rapid manufacturing of multi-layer mesh PVC gel structures [19].

To achieve the fabrication of a multilayer porous PVC gel actuator via integrated printing, the key lies in finding electrode materials with high conductivity that can be effectively combined with a PVC core, allowing voltage to be applied to the PVC gel. Direct writing is an additive manufacturing technology that has attracted significant attention and has recently been used to print stretchable sensors [15], flexible electronic devices [21], ferromagnetic elastomers [22], soft actuators [23], and soft robotics devices [24]. Utilizing direct writing technology in manufacturing PVC gel artificial muscles can address the challenge of integrated printing of an electrode and core on a single platform. Direct writing has been successfully combined with electrode printing using curable conductive solutions during the printing process [25].

In this study, we devised and demonstrated a novel approach of using fully automatic multi-material direct writing to achieve integrated printing of multilayer porous PVC gel artificial muscles. Carbon nanotube-doped silicone (CNT-PDMS) composite inks were developed for printing electrode layers of artificial muscles. The parameters of the electrode ink configuration, mesh, and planar electrode printing were optimized. The feasibility of printing an electrode was theoretically explored. Several experimental techniques were employed to evaluate the feasibility of the process, including conductivity tests, rheological measurements, and electro-mechanical tests.

## 2. Results and Discussion

### 2.1. Printability of CNT-PDMS and PVC Gel Inks

Proper rheology is essential for direct writing because direct writing relies on continuous deposition through an extrusion device. The printing ink should be not difficult to extrude under moderate pressure and should be able to maintain its shape when the shear stress is released, requiring a suitable rheology and a moderate elastic modulus to prevent collapse during and after extrusion.

**Figure 3:**
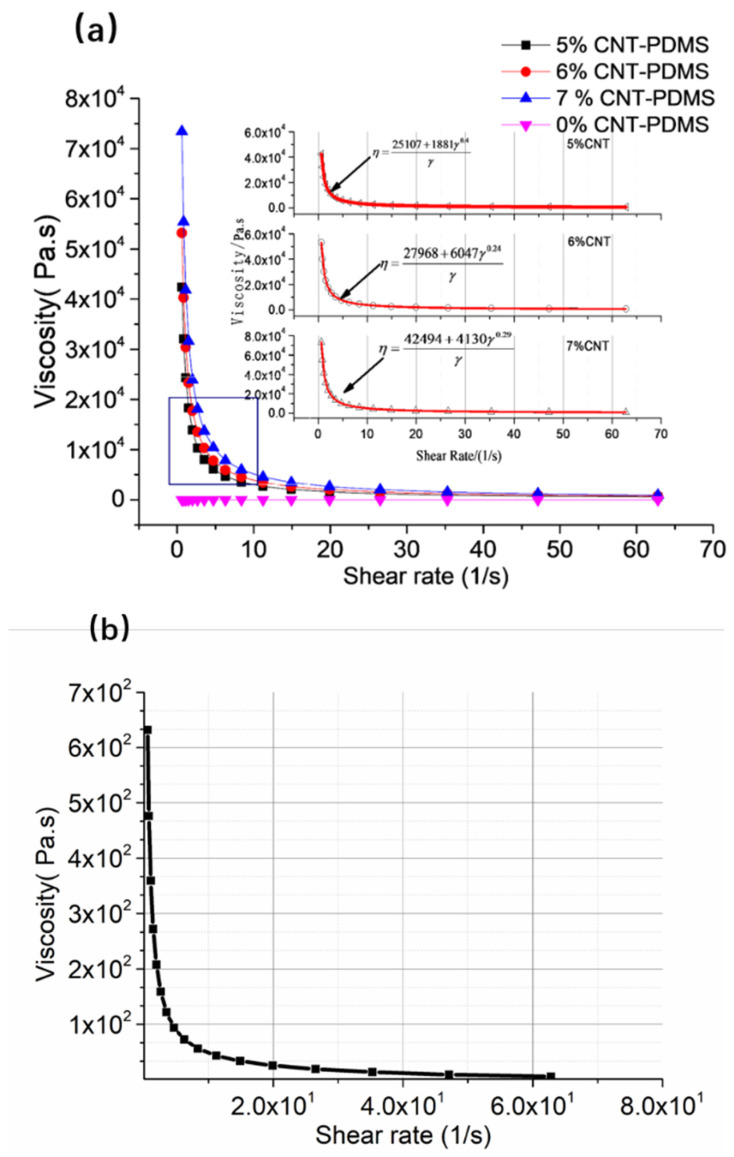
Shear rate dependence of viscosities of (**a**) CNT-PDMS inks and (**b**) PVC gel inks.

The CNT-PDMS and PVC gel inks with various compositions and ratios all exhibited shear thinning, as illustrated in Figure 3. The relationship between viscosity and shear rate can be fitted using the Herschel–Bulkley model [26,27,28]:

(1) $$\eta =\frac{\tau_{y}+K\gamma^{n}}{\gamma }=\frac{\tau_{y}}{\gamma }+K\gamma^{n-1}$$

where $\tau_{y}$ is the shear yield stress of the printing ink, *K* is the viscosity index of the printing ink, γ is the shear deformation rate, and *n* is the shear thinning index (*n* < 1).

Figure 3 shows that the viscosities of all printing inks decrease with shear rate, which meets direct writing requirements.

**Figure 4:**
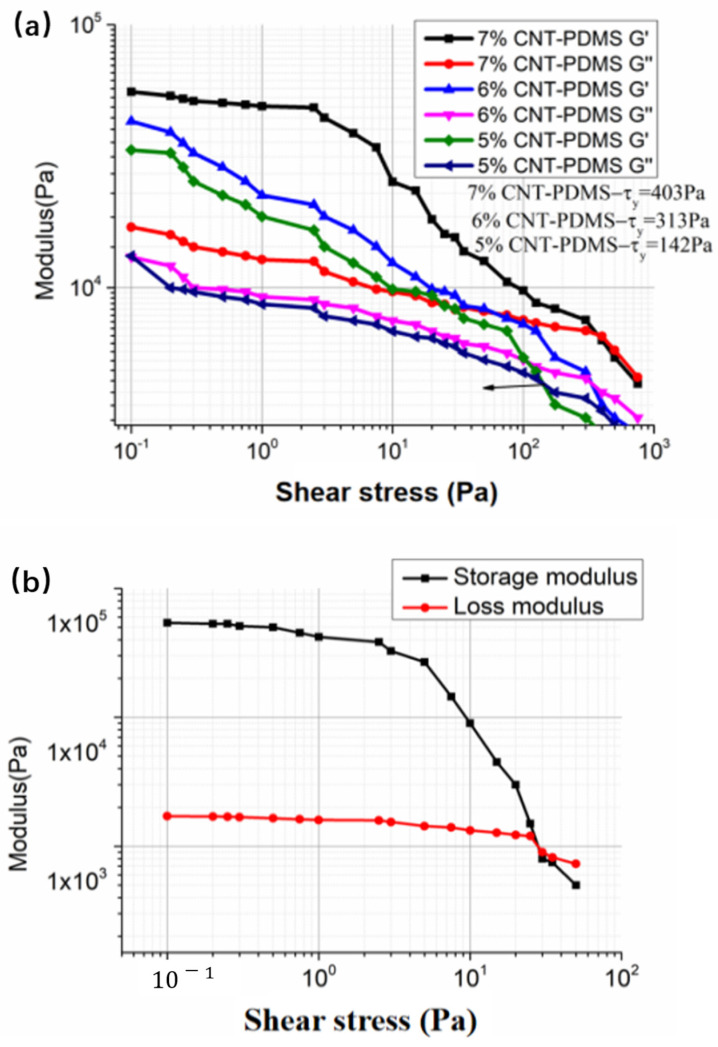
Plot of the $G^{\prime }$ and $G^{\prime \prime }$ dependence of shear rate for (**a**) CNT-PDMS inks and (**b**) PVC gel inks.

The elastic modulus $G^{\prime }$ and viscous modulus $G^{\prime \prime }$ significantly impact forming capacity. A distinctive feature of CNT-PDMS inks that facilitates direct writing is their ability to rapidly maintain their shape after shear stress is released, as shown in Figure 4. Each of these ink blots is difficult to extrude with a lower viscosity ($G^{\prime \prime }>G^{\prime }$) under a shear stress, but rapidly recovers its mechanical properties ($G^{\prime \prime }<G^{\prime }$) after the shear stress is reduced or removed. The yield stresses of CNT-PDMS inks in this study were found to increase with increasing carbon content. Previous studies have established that successful inks for printing spanning structures typically have $\tau_{y}>200\;\mathrm{Pa}$ and $G^{\prime }>10^{4}\;\mathrm{Pa}$ [24].

A high $\tau_{y}$ can prevent collapse of the lower layers of printed CNT-PDMS when it is loaded with the weight of the upper layers in a printed part, while a high G′ prevents elastic buckling of tall features and excessive sagging of spanning features [27]. Therefore, the CNT content of CNT-PDMS needs to exceed 6% to be suitable for printing, as shown in Figure 4a. The PVC gel ink demonstrates solid-like behavior ($G^{\prime \prime }<G^{\prime }$) at stresses from 0.1 to 29.81 Pa and liquid-like behavior ($G^{\prime \prime }>G^{\prime }$) at stresses above 29.81 Pa in Figure 4b, which indicates that this ink is suitable for direct writing. The difference between the elastic and loss moduli of the PVC gel ink is large enough ($\frac{G^{\prime }}{G^{\prime \prime }}=31\gg 1.25$), which indicates the ink configuration meets the shape retention condition [26].

**Figure 5:**
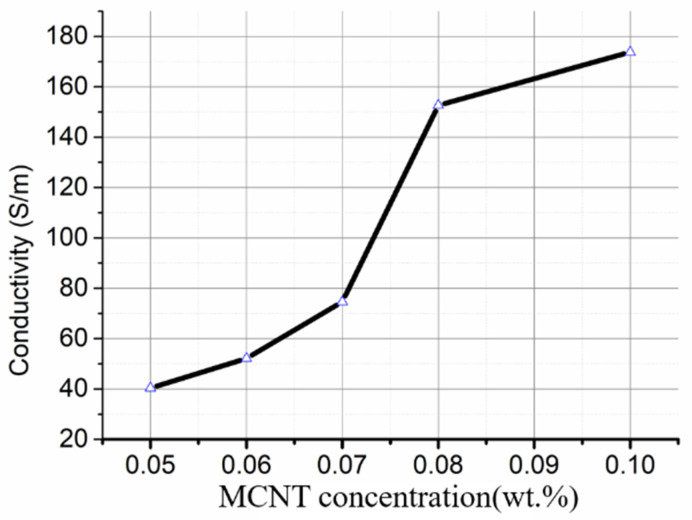
Effect of carbon content on conductivity of the CNT-PDMS composite inks.

We also studied the influence of CNT content on conductivity of CNT-PDMS composites. CNT-PDMS composite conductivity increases gradually with increasing CNT content, as shown in Figure 5. Increasing the CNT mass fraction from 6 wt% to 8 wt% sharply increases the conductivity of a CNT-PDMS composite. The conductivity of an electrode obviously satisfies the requirement (>60 s/m; the voltage loss tends to zero) when the carbon content is 6 wt% to 8 wt%.

### 2.2. CNT-PDMS Printed Spanning Structure

Shape fidelity depends not only on the rheological properties of inks, but also on the design of the printing structure and printing parameters. Printing a spanning structure electrode needs further theoretical analysis and experimentation. When CNT-PDMS composite ink is extruded from a nozzle with length *L* and diameter *D*, the stress in the radial direction can be calculated with [28].

(2) $$\tau_{r}=\frac{\left(P-P_{0}\right)}{2L}r$$

where *P* is the extrusion pressure, $P_{0}$ is the atmospheric pressure, and *r* is the radial distance from the extrusion nozzle center.

**Figure 6:**
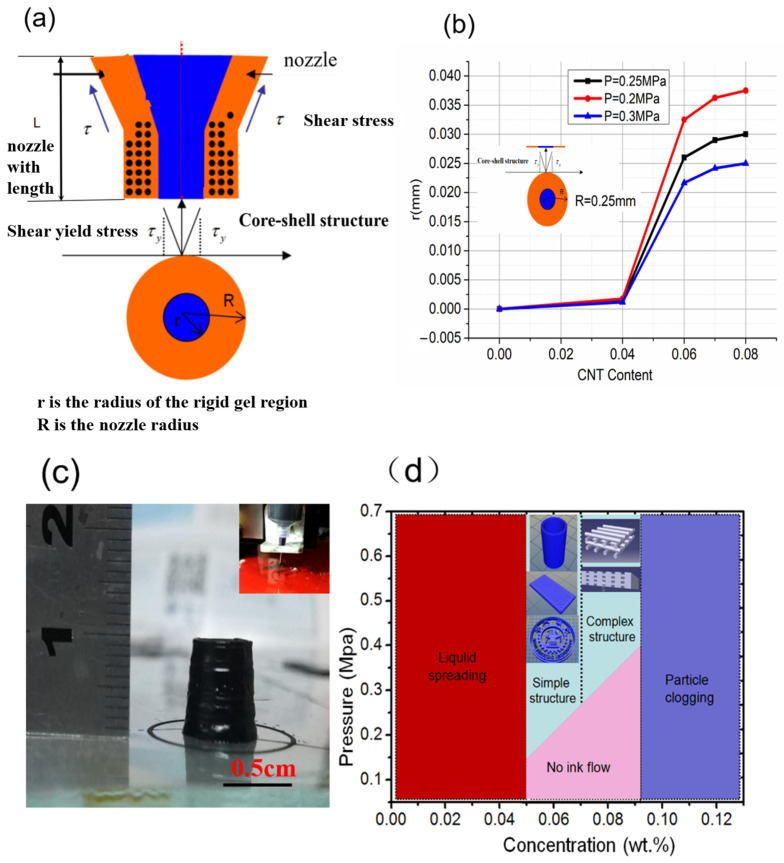
(**a**) Core-shell structure formed after extrusion of CNT-PDMS ink. (**b**) Radius of rigid gel region (*r*) increasing with increasing carbon content. (**c**) Extensional behavior of yield-stress fluids: 5%-CNT/PDMS keeps its shape, allowing a superimposed structure to be printed. (**d**) Phase diagram for printing a carbon nanotube-doped silicone electrode.

When $r<\frac{2L\tau_{y}}{\left(P-P_{0}\right)}$, the storage modulus $G^{\prime }$ of the printing ink in this region is higher than the loss modulus $G^{\prime \prime }$; this is the rigid gel region. When $r>\frac{2L\tau_{y}}{\left(P-P_{0}\right)}$, $G^{\prime }$ in this region is lower than $G^{\prime \prime }$; this is the region where it is difficult to maintain the shape. As long as $G^{\prime }>G^{\prime \prime }$, the phase state of the extruded filament diameter is the core-shell structure shown in Figure 6a. The core-shell size is different when the CNT-PDMS has different carbon contents. Figure 6b shows the core-shell size (*r*/*R*) distribution of CNT-PDMS printing inks with carbon content varying from 0 to 8 wt% after extrusion under pressures of 0.2 MPa, 0.25 MPa, and 0.3 MPa. The radius of the rigid gel region (*r*) increases with increasing carbon content. The rigid gel region is larger when the CNT-PDMS has higher carbon content, and the material has better shape retention. Inks with low carbon nanotube content (5 wt% and 6 wt%) allow the printed filaments to coalesce to form a monolithic structure without pores. This allows only simple stack structures to be printed, such as an XJTU badge and cylinders, as shown in Figure 6c. Filaments extruded from inks with higher CNT content (7 wt%, 8 wt%, and 9 wt%) maintained their extruded shape well, allowing the cross-gap features in Figure 6d to be printed. We found that when the CNT concentration was between 7 wt% and 9 wt% and the pressure was above a certain threshold, the ink could be printed into 3 D structures with gap-spanning features, while when the CNT concentration was below 5 wt% or above 9 wt%, the ink could not be printed owing to liquid diffusion or clogged nozzles, respectively. Furthermore, when the printing pressure was below the threshold, the ink could not flow out of the nozzles. Our modulation enabled the 7 wt% CNT/PDMS composite ink to obtain not only suitable rheological properties that ensured printing accuracy while avoiding nozzle clogging, but also suitable electrical conductivity for electrodes in PVC gel artificial muscles. Thus, 7 wt% CNT/PDMS composite ink was consistently used in the printing experiments.

The filament precision of printed filaments is also a key parameter in shaping 3D structure, and we controlled it by adjusting the 3D process parameters including the extrusion pressure and nozzle moving speed. For nozzle motion on a printed CNT-PDMS composite ink, the filament diameter *D* can be calculated via [27].

(3) $$D=\sqrt{\frac{2\left(P-\sigma +\rho gL\right)R^{4}}{\eta_{0}LV}}$$

where *P* is the pressure, *σ* is the average shear stress of the ink, *ρ* is the ink density, *R* is the nozzle radius, *η*_0_ is the zero-shear viscosity, and *V* is the nozzle moving speed.

**Figure 7:**
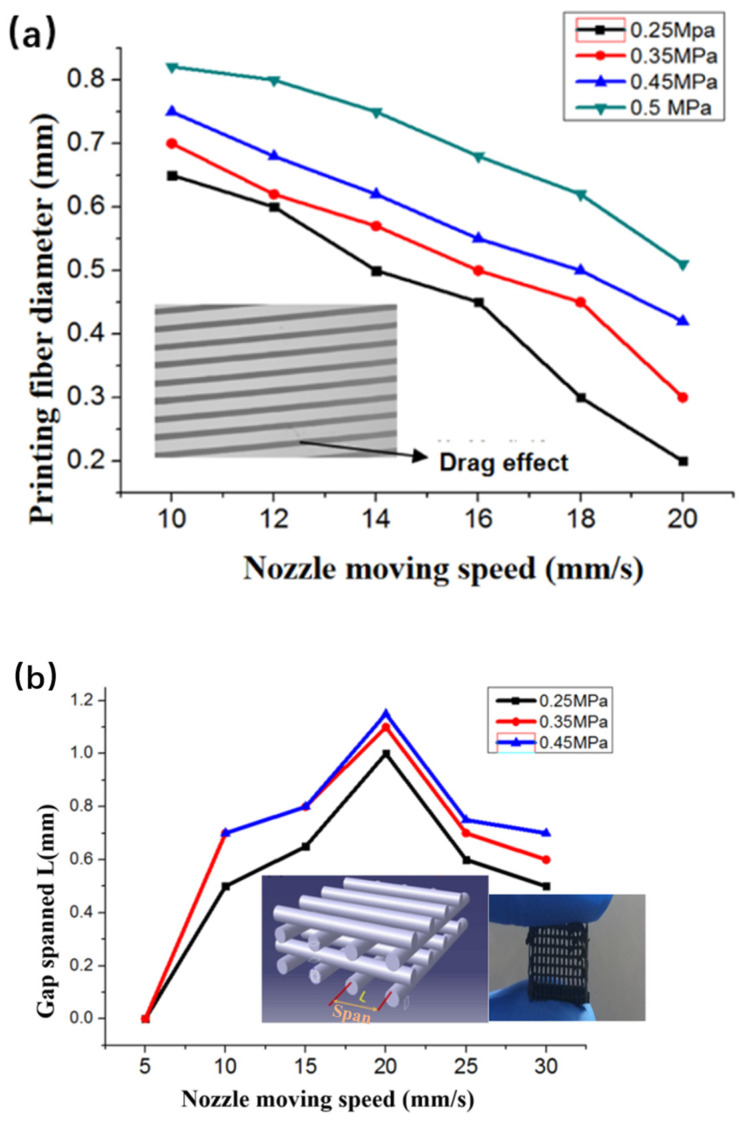
(**a**) Dependence of nozzle speed and extrusion pressure on filament diameter using 7 wt% CNT-PDMS. (**b**) Dependence of nozzle speed and extrusion pressure on maximum achievable printing span.

From the formula, the main factors affecting the filament diameter are extrusion pressure and nozzle speed. A filament accuracy of 0.2 mm was obtained when the print parameters were *P* = 0.25 MPa and *V* = 20 mm/s, as shown in Figure 7a.

As described in Section 4.1, printing multiple PVC gel actuators requires the ability to print spanning structure on a mesh electrode. Previous studies have shown that storage inks satisfy condition [28].

(4) $$G^{\prime }\geq 1.4\rho gs^{4}D$$

where *ρ* is the density of the printing ink, *D* is the ideal printed filament diameter, *s* is the standardized span ($S=\frac{L}{D}$), *L* is the span, and *g* is the acceleration of gravity.

For the CNT-PDMS composite spanning structure to be printed with *D* = 0.2 mm, *L* = 1 mm, and ρ=1.03 g/mm, the CNT-PDMS storage modulus should meet the following condition:

(5) $$G^{\prime }>1.4\rho gs^{4}D=1.4\times 1.03\times 9.8\times ({\frac{1}{0.2})}^{4}\times 0.2\;\mathrm{Pa}=1766.45\;\mathrm{Pa}$$

Therefore, the configured 7 wt% CNT-PDMS ink had the ability to print a spanning mesh structure.

Figure 7b shows the gap-spanning displacement (*L*) acquired at appropriate nozzle moving speeds using 7 wt% CNT-PDMS ink. *L* reached 1.1 mm at *P* = 0.25 MPa and *V* = 20 mm/s. The printed filaments easily collapsed at *V* < 10 mm/s and readily broke when *V* > 30 mm/s.

The scan spacing is the most critical factor affecting flatness when plane electrodes are printed. If the scanning space is too large, the overlaps between filaments are too small and even gaps appear, resulting in poor mechanical strength and reduced conductivity of the printing CNT-PDMS composite. If the scanning space is too small, the fibers accumulate in the height direction, and swell or even overflow owing to excessive accumulation of CNT-PDMS composite materials in the length and width directions, making it difficult to print the spanning structure.

**Figure 8:**
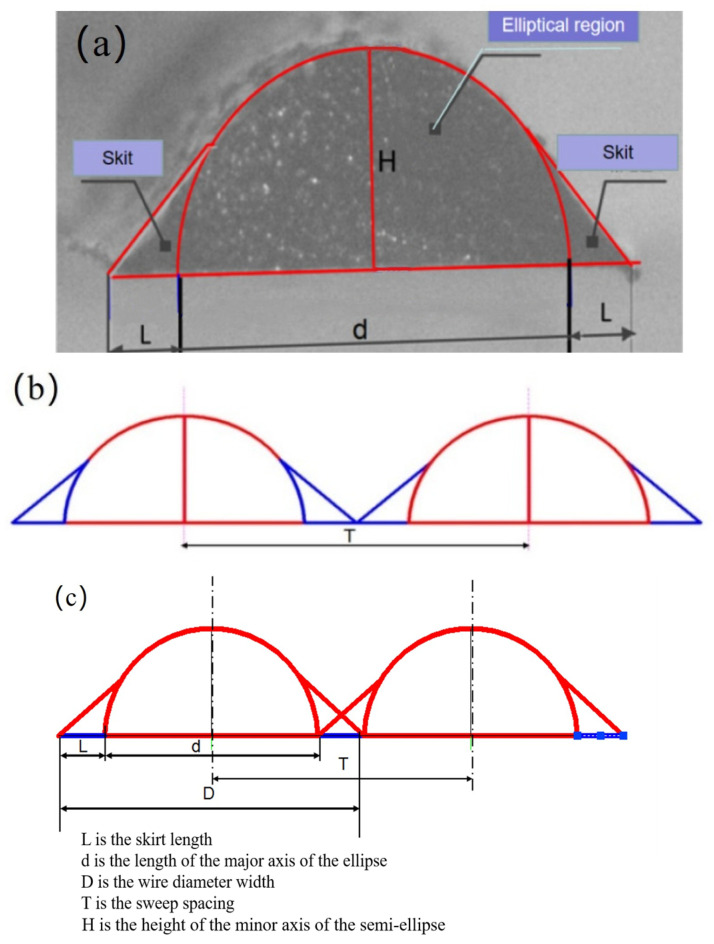
(**a**) Printed fiber under the microscope. (**b**) Schematic when the scan spacing is d+2L. (**c**) Schematic when the scan spacing is d+L.

Figure 8a shows a cross section of the filament with a diameter of 0.2 mm under the microscope. The cross section of the filament was divided into a semi-elliptical area and skirt area using AutoCAD 2018. The filament width is

(6) D=d+2L

where *d* is the length of the major axis of the ellipse, and *L* is the skirt length.

The ratio between dimensions *D*, *L*, and *H* is found to be *D*:*L*:*H* = 695:150:336 using the AutoCAD dimensioning function. When the scanning space *T* is equal to the wire diameter width d+2L, as shown in Figure 8b the filaments just overlap with the skirt, the printed plane does not perform well in terms of strength and conductivity. When the scan spacing *T* is less than *d*, the filaments that have been printed are greatly disturbed when the CNT-PDMS ink is extruded and pulled into the filaments. It was very easy for the filaments to collapse owing to the mutual disturbance between the lines during the printing of a mesh or span structure.

When the scan spacing *T* is d+L in Figure 8c, the overlap between filaments is only the skirt area, which not only ensures that the main elliptical area between the filaments does not interfere, but also ensures a good overlap rate. The ratio of scanning space *T* to printing wire diameter *D* is

(7) $$\frac{T}{D}=\frac{d+L}{d+2L}$$

The printing line width D=d+2L is 0.2 mm, so the scan spacing *T* is 0.166 mm.

**Figure 9:**
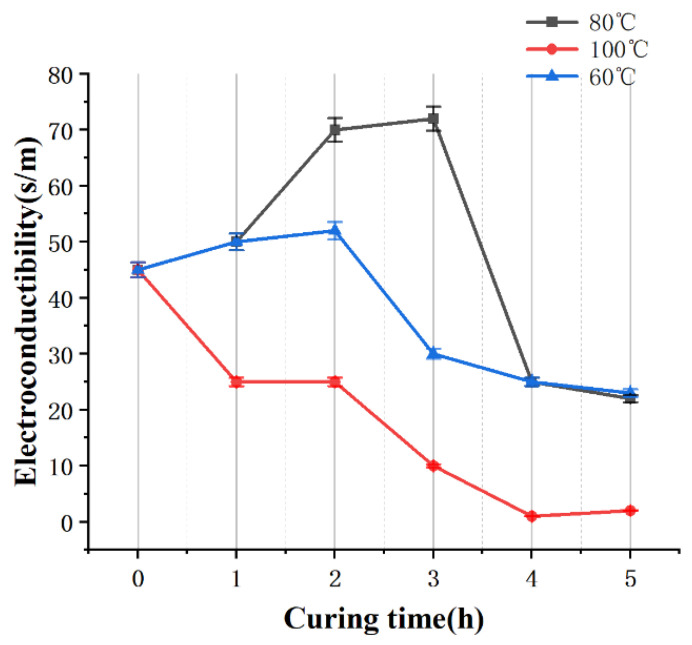
Conductivity of printed CNT-PDMS cured over different lengths of time at different temperatures.

After the CNT-PDMS structure is printed, an appropriate curing temperature and appropriate extension of curing time can improve the crosslinking of the polymer and promote formation of a conductive network. Figure 9 shows the conductivity of CNT-PDMS cured at different temperatures as a function of curing time. The optimum curing temperature of the printing material is 80 °C.

Table 1 shows the parameters for printing CNT-PDMS.

**Figure 10:**
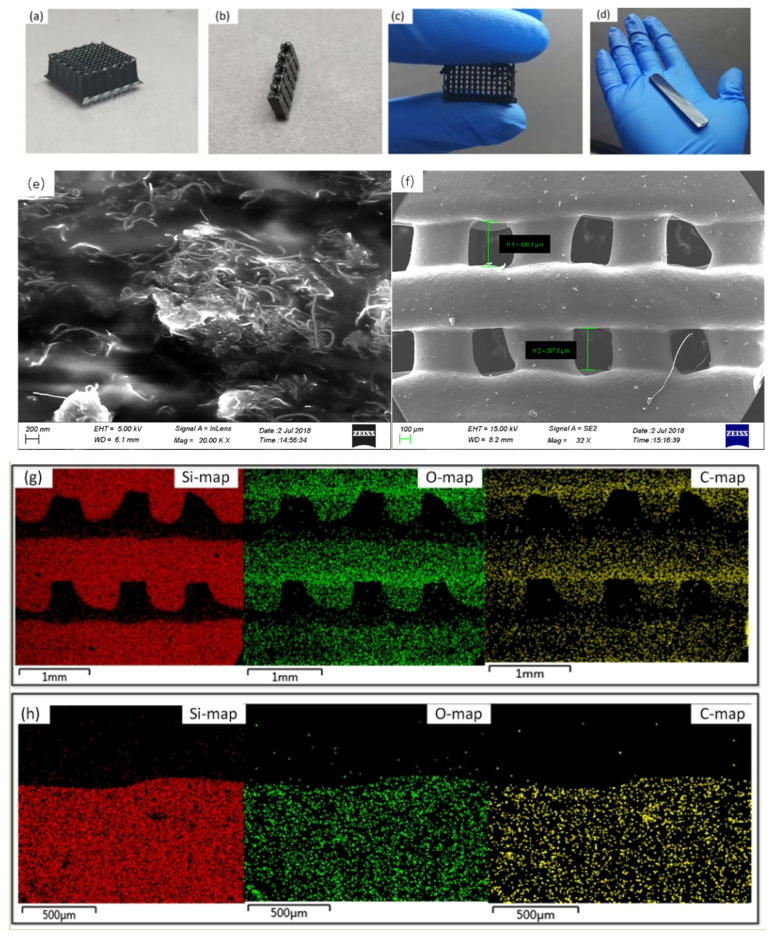
Maps of a printed CNT/PDMS composite. (**a**) Images of the printed mesh electrode structure. (**b**) Images of the printed 3D spanning feature. (**c**) Images of the printed multi-span structure. (**d**) Images of the printed gradient structure with variable porosity. (**e**) Cross sections of SEM images of the CNT/PDMS composite in the mesh electrode. (**f**) SEM of the printed 3D spanning feature. (**g**) EDS spectra and element maps of Si, O, and C (42.36% C, 19.35% O, and 38.29% C) for the mesh electrode. (**h**) EDS spectra and element maps of Si, O, and C for a gradient structure with variable porosity (40.87% C, 25.87% O, and 33.20% Si).

To demonstrate the ability to print complex structures in 7 wt% CNT-PDMS, various 3D electrode structures were printed, including a free-standing 3D mesh with hollow features (Figure 10a), 3D arch bridges with spanning gap properties (Figure 10b,c), and a gradient structure with porosity that varies with thickness (Figure 10d). The microstructures of the printed CNT/PDMS composites are shown in the fracture cross-sectional scanning electron microscope (SEM) image in Figure 10e. One can see that the carbon nanotubes are randomly and well distributed in the polymer matrix with a high aspect ratio, forming a conductive network in the matrix. Gap-spanning is observed in the SEM image in Figure 10f, which highlights the small feature size and spanning elements achieved by our direct-writing free-form fabrication of CNT/PDMS. Elemental analysis based on energy dispersive X-ray spectroscopy (EDS) showed three main components in the sample, silicon, oxygen, and carbon. Figure 10g,h show cross-sectional EDS images of the gap spanning structure and the wood pile structure. The carbon contents were calculated as being similar by comparing the EDS images of the two different parts and different structures, which proved that CNTs were randomly dispersed in silicone.

### 2.3. Printed Planar PVC Gel Structure

**Figure 11:**
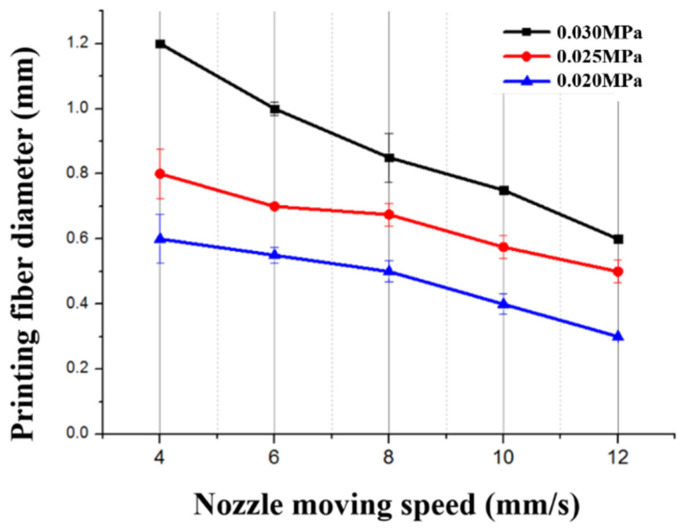
Influence of the extrusion rate and pressure on the printed diameter of PVC gel.

To choose the suitable printing parameters, we studied the effects of extrusion pressure P and nozzle moving speed V on the filament diameter. A minimum filament diameter for PVC gel ink could be achieved with *P* = 0.020 MPa and *V* = 12 mm/s, as shown in Figure 11.

### 2.4. Integrated Printing of Multilayer Porous PVC Gel

**Figure 12:**
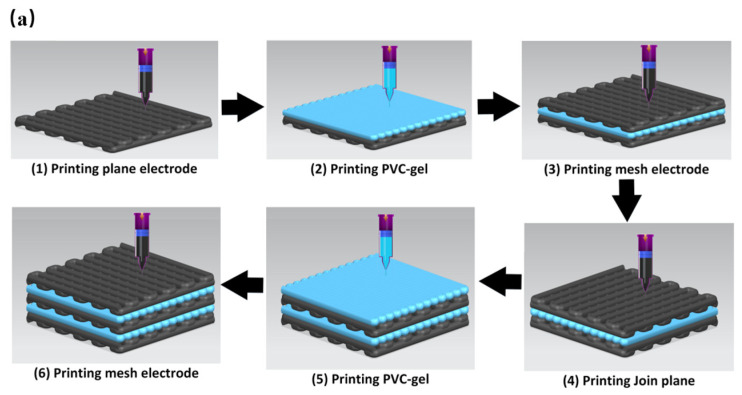

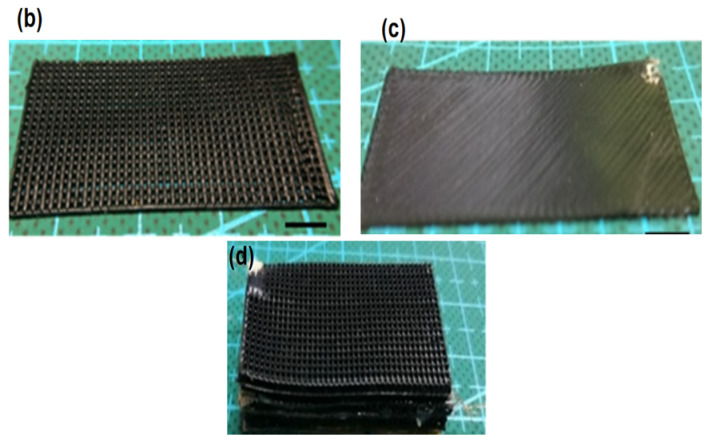
(**a**) Manufacturing process of integrated printing of PVC gel artificial muscle. (**b**) Printed CNT-PDMS composite mesh positive pole. (**c**) Planar negative pole. (**d**) Multilayer PVC gel artificial muscle obtained via integrated printing.

On this basis, we studied integrated multimaterial printing of a stacked porous PVC gel artificial muscle, as shown in Figure 12a. This process involved printing a CNT-PDMS composite planar negative pole, followed by printing the PVC gel core layer and finally printing a CNT-PDMS composite mesh positive pole on top, thus achieving integrated printing of an actuating unit of a PVC gel artificial muscle. Figure 12b,c show the successfully printed CNT-PDMS composite electrodes with 80-mesh positive poles and a planar negative pole. A three-unit PVC gel structure was manufactured by repeating the process of Figure 12a, as shown in Figure 12d.

### 2.5. Performance Testing and Discussion

**Figure 13:**
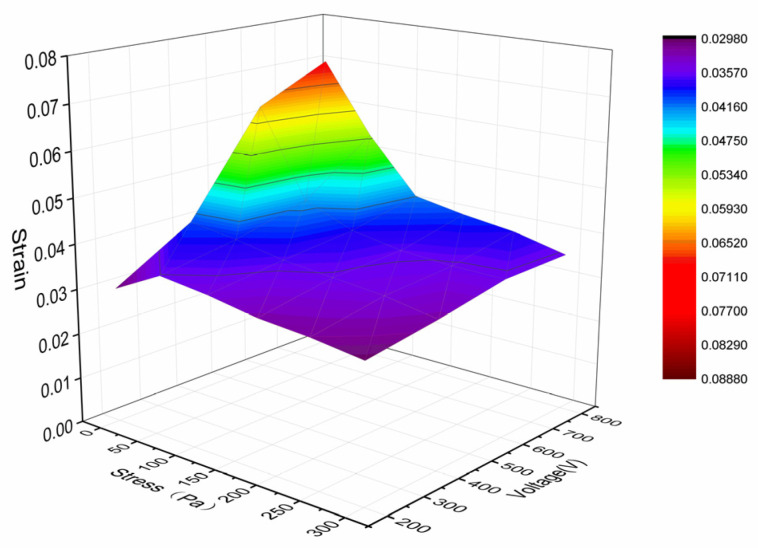
Performance of the printed planar PVC gel actuator.

The printed multilayer porous PVC gel actuator had wires and silver paste to connect the external voltage. The strain–voltage relationship of the actuator without load was measured at 1 Hz from 200 to 800 V with a 50% duty cycle square wave, and its strain–load relationship was measured with a load M = 0–70 g under a constant voltage of 800 V at 1 Hz with a 50% duty cycle square wave. The results are shown in Figure 13. The strain of the actuator reached 7% at 800 V.

The deformation of the printed actuator can be seen in the Video S1 in the Supplementary Material. This result shows that it is feasible to manufacture a whole stacked porous PVC gel artificial muscle fully automatically via integrated printing.

## 3. Conclusions

In this study, we printed CNT-PDMS for manufacturing multilayer porous PVC gel artificial muscle via direct writing. Inks with modulated rheological properties satisfied the needs of the electrode, core material, and structure. The parameters for printing planar and mesh electrodes were explored theoretically and experimentally. Performance testing of the printed PVC gel actuators showed the feasibility of direct writing. Our flexible PVC gel artificial muscles prepared through integrated printing have good actuating performance, and have the potential to be applied in human–machine interfaces, soft robotics, medical rehabilitation, flexible electronics, and other fields. The strategy that we developed here opens a simple and practical way to overcome the shortcomings of the traditional manufacturing methods, and will make it possible for PVC gel to be widely used in many fields.

## 4. Materials and Methods

### 4.1. Designing and Manufacturing PVC Gel Artificial Muscle

**Figure 14:**
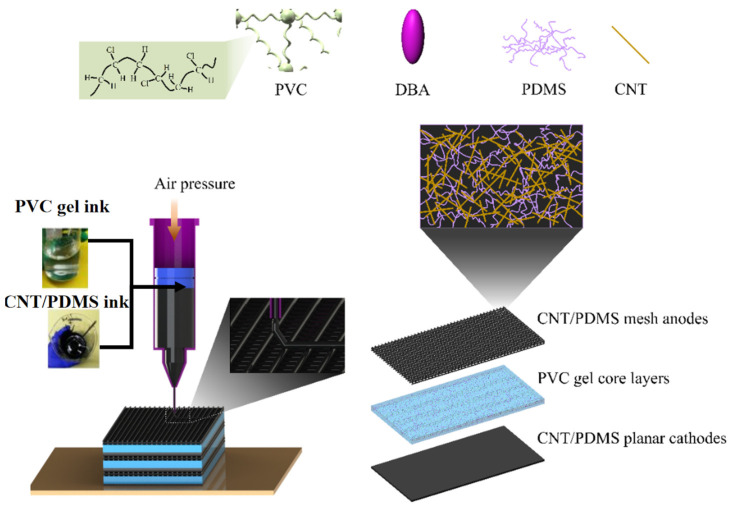
Schematic of the process for integrated direct writing of porous PVC gel artificial muscles.

A multilayer porous PVC gel artificial muscle comprises a series of superimposed actuating units, each of which consists of a planar negative pole, a planar PVC gel core layer, and a mesh positive pole, as illustrated in Figure 14. CNT-PDMS composite inks were used to print mesh and planar electrodes to connect the PVC core layer. Printing multiple porous PVC gel actuators requires planar printing capabilities on a grid, which means CNT-PDMS requires the ability to print spanning structures. In this way, we printed various functional layers with different materials and structures and achieved integrated direct writing of multiple porous PVC gel actuators.

### 4.2. Material System

The materials and reagents used in the experiment included PVC (polymerization degree of 4000, Scientific Polymer Products Inc., Ontario, NY, USA), DBA (Hubei Jusheng Technology Co., Ltd., Tianmen, China), THF (Tianjin Fuyu Fine Chemical Co., Ltd., Tianjin, China), multi-walled CNTs (TSW3, >98%, Time Nano, Beijing, China) with diameters of 10–20 nm and lengths of 0.5–2 µm, PDMS (Sylgard 184, Dow Corning, Midland, MI, USA), and isopropyl alcohol (IPA, >99%, Sigma-Aldrich, St. Louis, MO, USA).

The PVC gel ink was prepared by mixing PVC, DBA, and THF in a mass ratio of 1:7:12. The mixture was stirred with a magnetic stirrer for 2 to 3 days until a uniformly mixed viscous transparent gel was obtained [20].

To prepare the CNT-PDMS composite ink, multi-walled CNTs were first added to IPA and uniformly dispersed ultrasonically. PDMS-A was then added, and the mixed solution was again dispersed ultrasonically. Afterward, the mixed solution was concentrated by magnetic stirring (600 rpm) and heating (80 °C) for about 12 h to fully volatilize the IPA and obtain a mud-like composite. At this time, PDMS-B was added to achieve a mass ratio of PDMS-A: PDMS-B of 10:1, and the composite material was mixed by hand. Thus, we obtained the 3D-printable CNT-PDMS composite ink.

### 4.3. Ink Characterization

The rheological behaviors of the CNT-PDMS and PVC-gel inks were obtained at 30 °C using an electromagnetic rheometer (MCR 302, Anton Paar, Graz, Austria) fitted with a PP35Ti parallel plate with a diameter of 35 mm and gap of 0.5 mm. The viscosities of PVC gel ink, pure PDMS, and CNT-PDMS composite inks were measured as functions of shear rate, and their storage moduli and loss moduli were measured as functions of stress. Subsequently, the electrical properties of CNT-PDMS composites were characterized. CNT-PDMS composites with CNT contents of 5 wt%, 6 wt%, 7 wt%, 8 wt%, and 10 wt% were fabricated into long strips (40 mm × 12.5 mm × 1 mm) and then heated at 80 °C for 2 h to fully cure. We then used a source meter (2450 SourceMeter, Keithley, Tektronix, Cleveland, OH, USA) to measure the resistances of these specimens to calculate their conductivities.

### 4.4. Printing Process

In this study, we exemplified the printing of an 80-mesh electrode and bridge. Direct writing was conducted using a 3D printing system with multiple nozzles (Unibuliuder-D300, Yabao Jiaxing Technology Co., Ltd. Zhejiang, Jiaxing, China). First, the PVC gel structures were designed using 3D CAD software (SolidWorks 2016), which generated G codes for the conceptual structures. Second, a syringe filled with liquid printing material was installed in the designed position in the direct ink writing system. Finally, the printer produced the designed structure layer by layer according to the instructions in the G codes, simultaneously extruding the ink from the syringe at the specified extrusion rate under appropriate air pressure.

### 4.5. Performance Test of Artificial Muscles

**Figure 15:**
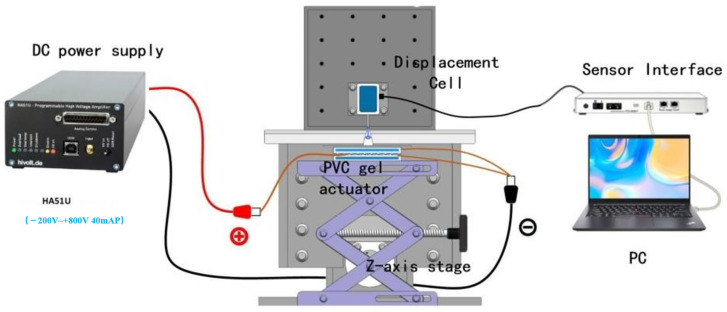
Schematic of the system used to test the performance of the planar PVC gel.

The performance of the 3D-printed artificial muscle was evaluated using the system depicted in Figure 15. A drive voltage was applied to the electrodes of the PVC gel muscle by combining an arbitrary waveform generator (DG4062, RIGOL, Suzhou, China) with a high-voltage power amplifier (HASIU, TREK, München, Germany). The artificial muscle deformed under the voltage, and its displacement was measured using a laser sensor.

## Figures and Tables

**Table 1:** Fixed printing parameters.

Moving Speed	Air Pressure	Scanning Spacing	Layer Height	Curing Temperature and Time
20 mm/s	0.25 MPa	0.166 mm	0.155	80 °C, 2 h

## Data Availability

The data that support the findings of this study are available from the corresponding authors upon reasonable request.

## Supplementary Materials

The following supporting information can be downloaded at: https://www.mdpi.com/article/10.3390/gels10070416/s1, Video S1: The deformation of the printed actuator.
